# Error-state Kalman filter for lower-limb kinematic estimation: Evaluation on a 3-body model

**DOI:** 10.1371/journal.pone.0249577

**Published:** 2021-04-20

**Authors:** Michael V. Potter, Stephen M. Cain, Lauro V. Ojeda, Reed D. Gurchiek, Ryan S. McGinnis, Noel C. Perkins

**Affiliations:** 1 Department of Mechanical Engineering, University of Michigan, Ann Arbor, MI, United States of America; 2 M-Sense Research Group, University of Vermont, Burlington, VT, United States of America; University of Illinois at Urbana-Champaign, UNITED STATES

## Abstract

Human lower-limb kinematic measurements are critical for many applications including gait analysis, enhancing athletic performance, reducing or monitoring injury risk, augmenting warfighter performance, and monitoring elderly fall risk, among others. We present a new method to estimate lower-limb kinematics using an error-state Kalman filter that utilizes an array of body-worn inertial measurement units (IMUs) and four kinematic constraints. We evaluate the method on a simplified 3-body model of the lower limbs (pelvis and two legs) during walking using data from simulation and experiment. Evaluation on this 3-body model permits direct evaluation of the ErKF method without several confounding error sources from human subjects (e.g., soft tissue artefacts and determination of anatomical frames). RMS differences for the three estimated hip joint angles all remain below 0.2 degrees compared to simulation and 1.4 degrees compared to experimental optical motion capture (MOCAP). RMS differences for stride length and step width remain within 1% and 4%, respectively compared to simulation and 7% and 5%, respectively compared to experiment (MOCAP). The results are particularly important because they foretell future success in advancing this approach to more complex models for human movement. In particular, our future work aims to extend this approach to a 7-body model of the human lower limbs composed of the pelvis, thighs, shanks, and feet.

## Introduction

Human lower-limb kinematic measurements are critical for many applications including gait analysis, enhancing athletic performance, reducing or monitoring injury risk, augmenting warfighter performance, and monitoring elderly fall risk, among others [1–5]. Historically, most research studies are constrained to laboratory environments where camera-based motion capture systems (MOCAP) are used to measure body segment kinematics. A major disadvantage of laboratory-based studies is that experimental constraints (e.g., limited capture volume, artificial environment, and observers) may alter how subjects perform tasks, making it difficult to extrapolate results to unconstrained (real-life) environments [6,7]. Some research studies require continuous monitoring of human kinematics, rendering laboratory-based methods ineffective [8–10]. In addition, camera-based systems are relatively expensive, require long setup times, and require trained researchers [1,7]. Collectively, the above realities of laboratory-based experiments may significantly limit the findings and benefits of the research. Thus, there remains a strong motivation to advance the use of wearable sensors to measure human kinematics outside of the lab environment.

The most utilized sensors for mobile kinematic measurements are inertial measurement units (IMUs) which contain three-axis accelerometers and angular rate gyroscopes (with some designs also including magnetometers, GPS, barometers, or other sensors). The resulting measurements can be integrated and/or differentiated to estimate the kinematics of the body segments to which they are attached [11]. However, because of measurement noise and finite sampling rates, kinematic variables estimated via numerical integration are subject to integration drift errors. Consequently, accurate estimates of kinematics from (noisy) IMU measurements must also correct for drift errors [10–14].

In the context of estimating lower-limb human kinematics with IMUs, approaches that leverage known kinematic conditions and relationships demonstrate success in correcting integration drift errors in certain applications [15]. One well-known example is the zero-velocity update (ZUPT) method for computing three-dimensional trajectories of the feet [13,16]. This method uses the fact that, during human walking, the foot (and attached IMU) must be nearly still (zero-velocity) at some point during each stance phase to correct for drift in the estimated foot velocity. Other methods successfully estimate joint angles for single joints. For example, IMU-based knee joint angle algorithms capitalize on the fact that the knee often acts as a hinge joint [17,18].

In addition to single-segment or single-joint methods highlighted above, recent work for multi-segment or multi-joint systems shows progress towards describing the human lower limbs. While proprietary products for such analyses exist (e.g., Xsens MVN Link, Noraxon Ultium Motion), they incorporate unspecified assumptions (hence, unspecified limitations) which is especially pertinent because accuracy of IMU-based methods are often task-specific [15]. Independent validation studies of these systems confirm such task dependence and also reveal that accuracy varies significantly between specific joint angles [19–21]. These proprietary products also have significant cost and rely on product-specific IMU hardware or even specialized wearable suits. Thus, a significant need exists for validated and well-documented methods to advance future research and applications.

Several methods exist for estimating the kinematics of the human lower limbs using a 7-body representations of the human lower limbs constituting the feet, shanks, thighs and hip. Ahmadi et al. [22] utilize a ZUPT method to estimate ankle position trajectories and combine those with individual segment orientation estimates to yield estimated lower-limb kinematics for straight walking on level ground and stairs. Optimization ensures the joint angles conform to assumed ranges of motion. Results are validated via comparison with MOCAP measurements for short trials (six passes through a MOCAP volume) that may not fully expose the accumulation of (long-term) drift error. The results demonstrate strong correlations (R >0.94) for joint angles, but only those restricted to the sagittal plane. Teufl et al. [23] employ an iterated extended Kalman filter to estimate lower-limb kinematics and with root-mean-square (RMS) joint angle differences (all three axes) below 6 degrees relative to MOCAP measures. Additionally, their method estimates RMS stride length and step width differences of 0.04 and 0.03 meters, respectively, compared to MOCAP [24]. However, their algorithm assumes level-ground (to correct vertical drift and to identify zero-velocity update times), which renders it unsuitable for quantifying gait on general (unconstrained) terrain as often encountered outdoors. Collectively, the limitations of the studies reviewed above point to the need for a general algorithm that accurately estimates lower-limb kinematics over long trials (i.e., greater than five minutes) and without assumptions of terrain morphology.

This paper presents a novel error-state Kalman filter (ErKF) method for estimating lower-limb kinematics using data from wearable IMUs. We use this method to estimate three degree-of-freedom (DOF) joint angles, stride length and step width. The method presented here extends Sola’s formulation of the ErKF for a single IMU [12] to a multi-IMU formulation and also incorporates biomechanical measurement models to correct integration drift errors. In contrast to [22], the method is effective for long integration times (i.e., long trials). In contrast to [23], the method does not embed a level-ground assumption. As a first step towards developing a complete (7-body) model of the human lower limbs, we first consider herein an approximate 3-body mechanical model. Doing so enables careful formulation and study of all key modeling steps but within the context of a simpler model. Additionally, evaluation on this well-characterized mechanical model permits direct evaluation of the ErKF method without the confounding error sources associated with human subjects including uncertainties in joint center locations, joint axes, sensor-to-segment alignment parameters, increased joint complexity, and soft tissue artefacts. Thus, evaluation of this novel ErKF method on a well-characterized 3-body mechanical model (a “walker”) is a critical step towards extension to a full (7-body) model of the human lower limbs. We demonstrate the success of the method by comparison to two reference data sets. In the first comparison, estimated kinematic variables are compared to ground truth obtained by simulation. In the second comparison, estimated kinematic variables are compared to those measured by MOCAP using an engineered 3-body walker.

## Methods

### 3-body lower-limb model

**Fig 1:**
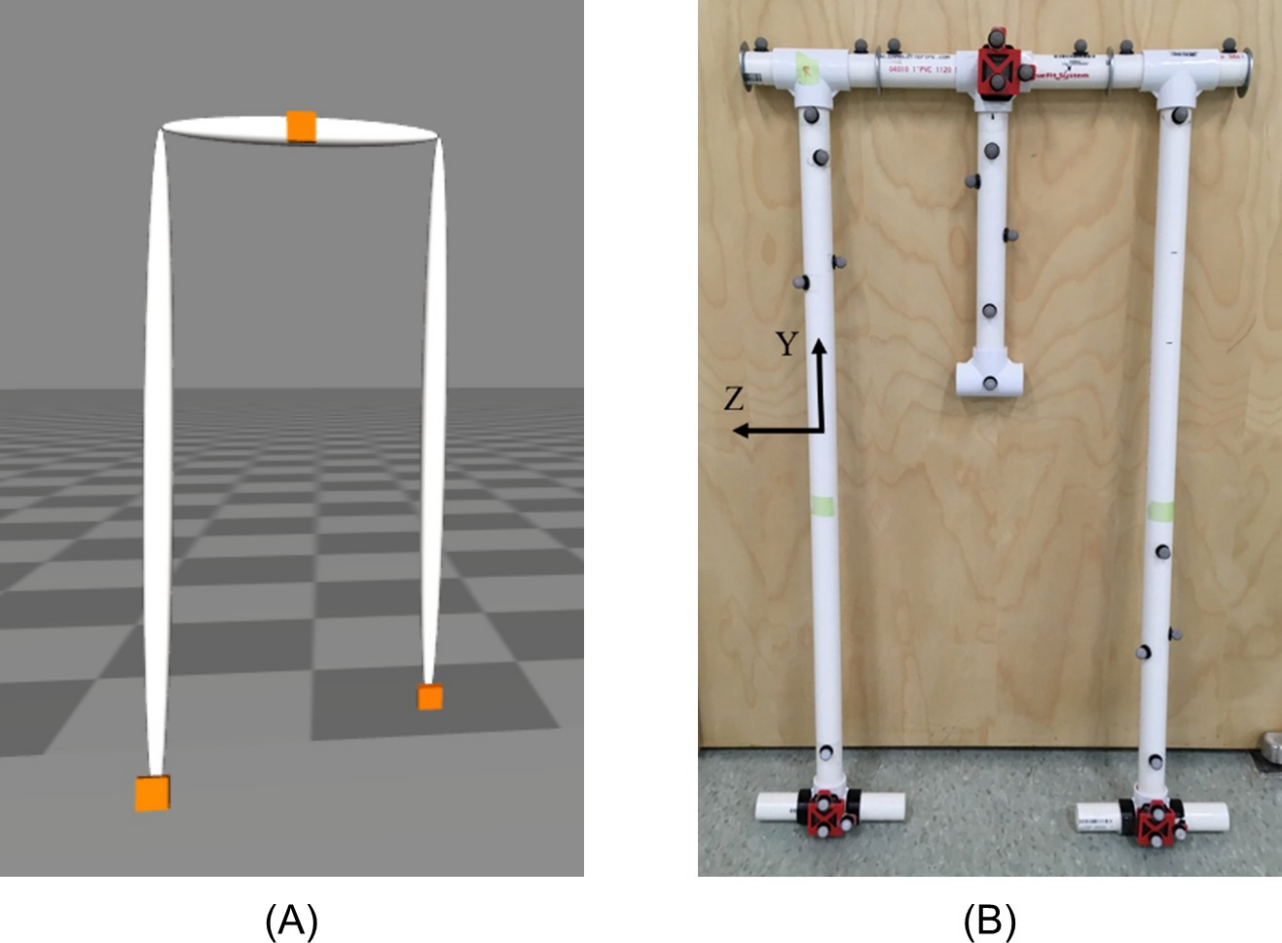
3-body model of the lower limbs. 3-body model of the lower limbs for (A) simulation and (B) experiment including IMU and reflective marker placement. Body-fixed axes defined such that the x-axis points anteriorly (not shown), the y-axis superiorly, and the z-axis to the right (aligned with hinge joint axis) when the model is in a neutral upright pose (as in (b)) for all limbs.

As a step towards estimating the lower-limb kinematics of a human (i.e., a 7-body model), we employ a novel ErKF method on a simplified (3-body) model of the lower-limbs for walking (i.e., a “walker”). We utilize the ErKF to estimate the poses (positions and orientations) of each IMU attached to each segment of the model. This simplified model embeds the key challenge to accurate lower-limb kinematic estimation from body-worn IMU data; namely, utilizing well-conceived measurement models to correct integration drift errors. The simplified model consists of a pelvis and two legs attached to the pelvis by hinge joints. The leg lengths and pelvic width are comparable to human anthropometrics (0.92 m and 0.39 m respectively). This model is simulated in OpenSim [25] (Fig 1A) and also fabricated for experiments (Fig 1B). The OpenSim model is modified from OpenSim’s "Dynamic Walking Challenge" example [26].

The model includes three IMUs with one near the center of mass of the pelvis (i.e., a sacrum-mounted IMU) and one at the distal end of each leg (i.e., foot-mounted IMUs). The IMUs are attached via a weld joint in the OpenSim model and via athletic tape in the experiment.

### Error-state Kalman filter method (ErKF)

While traditional extended Kalman filter equations are written with respect to the states of the system directly, the ErKF equations are written with respect to the errors in these states which then correct the estimated states. The ErKF demonstrates superior performance over a traditional extended Kalman filter for similar applications for aircrafts and robots due to key advantages including linearity, singularity avoidance, and simplicity [12,27–29]. Recent work also shows great promise for using ErKF formulations to improve the accuracy of joint angle estimates for biomechanical applications [30].

In this formulation, each segment is treated as a free body (i.e., possesses six DOF) which yields superior estimates of joint angles versus a minimal DOF kinematic chain, as previously illustrated for the human arm [31]. This algorithm requires knowledge of the positions of the joint centers and the directions of the anatomical axes for each segment in the attached IMU’s reference frame. We refer to these relationships as the sensor-to-segment alignment and assume they remain constant throughout a trial. Through the process model of the ErKF, IMU data from each body segment is integrated to estimate the time-dependent pose of the IMU (thus the time-dependent pose of the segment). Errors are corrected through known kinematic constraints (e.g., the joints between segments) and kinematic states (e.g., if a segment is momentarily at rest) that are incorporated in the measurement model. We describe the filter below.

States and error-states. This multi-IMU formulation for the ErKF draws from and extends Sola’s formulation for a single IMU [12] which also provides detailed derivations of relations used below. To reduce the size of the state, we do not estimate sensor biases or the gravitational acceleration. Instead, we assume sensor biases and the gravitational acceleration are well characterized (and constant) and these are estimated using IMU data during an intentional still period at the start of a trial. Therefore, the state for the *j*^*th*^ IMU, *x*_*j*_, is the (10x1) vector

$$x_{j}=\left[\begin{array}{c} p_{j}\\ v_{j}\\ q_{j} \end{array}\right]$$ (1)

where *p*_*j*_ is the (3x1) position vector of the accelerometer within the IMU in a world (i.e. lab-fixed) frame, *v*_*j*_ is the (3x1) velocity vector of the same point, and *q*_*j*_ is the (4x1) quaternion rotation vector (using Hamiltonian convention) that relates the IMU sense axis frame (hereafter called the IMU frame) to the world frame. In particular, we define *q* as the rotation quaternion that transforms a vector in the body-fixed frame (*y*^*b*^) to its components in the world frame (*y*^*w*^) per

$$\left[\begin{array}{c} 0\\ y^{w} \end{array}\right]=q⊗\left[\begin{array}{c} 0\\ y^{b} \end{array}\right]⊗q^{*}$$ (2)

where ⊗ denotes quaternion multiplication and q^*^ denotes the quaternion inverse.

The error state for the *j*^*th*^ IMU, *δx*_*j*_, is the (9x1) vector

$$\delta x_{j}=\left[\begin{array}{c} \delta p_{j}\\ \delta v_{j}\\ \delta \theta_{j} \end{array}\right]$$ (3)

where *δp*_*j*_ and *δv*_*j*_ denote errors in the position and velocity, respectively, and *δ*θ _*j*_ is the (three-component) attitude error vector (assumed to be small) defined such that the quaternion error *δq*_*j*_ obeys

$$\delta q_{j}=\left[\begin{array}{c} cos\left(\frac{\|\delta \theta_{j}\|}{2}\right)\\ bsin\left(\frac{\|\delta \theta_{j}\|}{2}\right) \end{array}\right]\approx [\begin{array}{c} 1\\ \frac{\delta \theta_{j}}{2} \end{array}].$$ (4)

where *b* is the unit vector in the direction of *δ*θ _*j*_ (i.e., the axis of rotation) and ‖·‖ is the Euclidean vector magnitude. The full state *x* is the concatenation of the states of all *n* IMUs in the system, namely

$$x=[\begin{array}{c} x_{1}\\ x_{2}\\ \vdots \\ x_{n} \end{array}].$$ (5)

Similarly, the full error state *δx* is

$$\delta x=[\begin{array}{c} \delta x_{1}\\ \delta x_{2}\\ \vdots \\ \delta x_{n} \end{array}].$$ (6)

The error-state covariance matrix (associated with the full error state) is denoted by *P*.

Process model. The prediction step of the ErKF uses the process model

$${\hat{x}}_{j,k+1}=f(x_{j,k},u_{j,k})$$ (7)

for each IMU where ${\hat{x}}_{j}$ denotes the prediction of *x*_*j*_, the additional subscript *k* denotes the *k*^*th*^ time-step, and *u*_*j*_ denotes the IMU data (acceleration and angular velocity). The state of each IMU at time-step *k+1* is then predicted from the state and IMU data at the previous time-step *k* per

$${\hat{x}}_{j,k+1}=\left[\begin{array}{c} p_{j,k}+v_{j,k}\Delta t+1/2(R_{j,k}a_{j,k}+g)\Delta t^{2}\\ v_{j,k}+(R_{j,k}a_{j,k}+g)\Delta t\\ q_{j,k}⊗\left[\begin{array}{c} cos\left(\|\omega_{j,k}\|\frac{\Delta t}{2}\right)\\ \frac{\omega_{j,k}}{\left\|\omega_{j,k}\right\|}sin\left(\|\omega_{j,k}\|\frac{\Delta t}{2}\right) \end{array}\right] \end{array}\right]$$ (8)

where *Δt* is the sampling period of the IMU, *R* is the rotation matrix corresponding to *q*, *g* is the gravitational acceleration vector (in the world frame), *a*_*j*_ is the acceleration measured by the *j*^*th*^ IMU, and *ω*_*j*_ is the angular rate measured by the *j*^*th*^ IMU. Note that the predicted state for each IMU (Eq 8) is independent of the other IMUs (each IMU is treated as an independent six DOF rigid body).

During the prediction step, the covariance matrix *P* is also estimated as follows. The Jacobian of the process model for the *j*^*th*^ IMU at time-step *k* with respect to its error state vector, *F*_*xj*,*k*_, follows from

$$F_{xj,k}=\left[\begin{array}{ccc} I_{3\times 3} & {\Delta tI}_{3\times 3} & 0_{3\times 3}\\ 0_{3\times 3} & I_{3\times 3} & -R_{j,k}{\left[a_{j,k}\right]}_{x}\Delta t\\ 0_{3\times 3} & 0_{3\times 3} & {\left(S\{\omega_{j,k}\Delta t\}\right)}^{T} \end{array}\right]$$ (9)

where *I*_*m×m*_ represents an *m × m* identity matrix, *0*_*m×m*_ represents an *m × m* matrix of zeros, the superscript *T* denotes the transpose of a matrix, *[y]*_*x*_ corresponds to the skew-symmetric form of *y*

$${\left[\begin{array}{c} y_{1}\\ y_{2}\\ y_{3} \end{array}\right]}_{x}=\left[\begin{array}{ccc} 0 & {-y}_{3} & y_{2}\\ y_{3} & 0 & {-y}_{1}\\ {-y}_{2} & y_{1} & 0 \end{array}\right]$$ (10)

and *S{w}* applies the Rodrigues’ rotation formula on the vector *w*

$$S\{w\}=S\{\varphi s\}=I_{3\times 3}cos(\varphi )+sin(\varphi ){\left[s\right]}_{x}+{ss}^{T}(1-cos(\varphi )).$$ (11)

Here, *φ* is the scalar magnitude of *w* and *s* is the unit vector in the direction of *w*. Due to the independence of the IMUs in the prediction step (Eq 8), the Jacobian of the full system process model relative to the error state at time-step *k*, *F*_*x*,*k*_, is

$$F_{x,k}=blkdiag(F_{x1,k},F_{x2,k},\ldots ,F_{xn,k})$$ (12)

where *blkdiag* denotes the block diagonal matrix composition. The process noise covariance for the *j*^*th*^ IMU, *Q*_*j*_, is

$$Q_{j}=\left[\begin{array}{ccc} 0_{3\times 3} & 0_{3\times 3} & 0_{3\times 3}\\ 0_{3\times 3} & \sigma_{a}^{2}{\Delta t}^{2}I_{3\times 3} & 0_{3\times 3}\\ 0_{3\times 3} & 0_{3\times 3} & \sigma_{\omega }^{2}{\Delta t}^{2}I_{3\times 3} \end{array}\right]$$ (13)

where $\sigma_{a}^{2}$ and $\sigma_{\omega }^{2}$ are the noise variances for the acceleration and angular rate signals, respectively, with the values being obtained from the manufacturer or through experiment. Note that this matrix is assumed constant for each IMU and thus we do not denote a time step. Again, due to the independence of the IMUs in the prediction step, these matrices can be concatenated to form the full system process noise covariance matrix, *Q*, per

$$Q=blkdiag(Q_{1},Q_{2},\ldots ,Q_{n}).$$ (14)

Thus, the prediction update for *P* is

$${\hat{P}}_{k+1}=F_{x,k}P_{k}F_{x,k}^{T}+Q.$$ (15)

The process model equations above detail predictions for the state and the error-state covariance. Note that the estimated error-state mean is not calculated because it is always zero throughout the process model because the error-state mean is initialized to zero and is reset to zero following any measurement update.

Measurement model. In the absence of any measurement during this time step we use the predicted state and error-state covariance as the best estimates at time-step *k+1*, namely

$$x_{k+1}={\hat{x}}_{k+1}$$ (16)

$$P_{k+1}={\hat{P}}_{k+1}.$$ (17)

However, when measurements are available during this time step, we apply corrections using the measurement model to improve the estimates as follows. The measurement model takes the functional form

z=h(x)+c (18)

where *z* is the observed measurement, *h(x)* is the expected measurement represented as a function of the state *x*, and *c* is Gaussian white noise with covariance *C*. Specific measurement models follow below. For each, we linearize the measurement equation by defining the Jacobian *H* evaluated at *x*

$$H={\frac{\partial h}{\partial \delta x}|}_{x}.$$ (19)

Consistent with [12], we use the chain rule to decompose *H* as

$$H={\frac{\partial h}{\partial x}|}_{x}{\frac{\partial x}{\partial \delta x}|}_{x}=H_{x}X_{\delta x}$$ (20)

where *H*_*x*_ depends on the measurement model and *X*_*δx*_ depends only on the estimated orientation. Next, the Kalman gain, *K*, and error-state mean, $\hat{\delta x}$ are updated per

$$K_{k}={\hat{P}}_{k+1}H_{k}^{T}{\left(H_{k}{\hat{P}}_{k+1}H_{k}^{T}+C_{k}\right)}^{-1}$$ (21)

$${\hat{\delta x}}_{k+1}=K_{k}(z_{k}-h({\hat{x}}_{k+1})).$$ (22)

The error-state mean for each IMU, ${\hat{\delta x}}_{j}$, updates its respective nominal state per

$$x_{j,k+1}=\left[\begin{array}{c} {\hat{p}}_{j,k+1}+{\hat{\delta p}}_{j,k+1}\\ {\hat{v}}_{j,k+1}+{\hat{\delta v}}_{j,k+1}\\ {\hat{q}}_{j,k+1}⊗\left[\begin{array}{c} cos(\left\|{\hat{\delta \theta }}_{j,k+1}\right\|/2)\\ \frac{{\hat{\delta \theta }}_{j,k+1}}{\left\|{\hat{\delta \theta }}_{j,k+1}\right\|}sin(\left\|{\hat{\delta \theta }}_{j,k+1}\right\|/2) \end{array}\right] \end{array}\right]$$ (23)

where

$${\hat{\delta x}}_{j}=[\begin{array}{c} {\hat{\delta p}}_{j}\\ {\hat{\delta v}}_{j}\\ {\hat{\delta \theta }}_{j} \end{array}].$$ (24)

After the nominal state mean is updated, the error-state mean is reset to zero and the error-state covariance is updated per

$$P_{k+1}=G_{k}(I_{9n\times 9n}-KH){\hat{P}}_{k+1}G_{k}^{T}$$ (25)

where *G*_*k*_ is the Jacobian of the error-state reset operation with respect to the error state at time-step *k*, defined as

$$G_{k}=blkdiag(G_{1,k},G_{2,k},\ldots {,G}_{n,k})$$ (26)

where

$$G_{j,k}=[\begin{array}{ccc} I_{3\times 3} & 0_{3\times 3} & 0_{3\times 3}\\ 0_{3\times 3} & I_{3\times 3} & 0_{3\times 3}\\ 0_{3\times 3} & 0_{3\times 3} & I_{3\times 3}-{[\frac{1}{2}{\hat{\delta \theta }}_{j,k+1}]}_{x} \end{array}].$$ (27)

We note that the addition of the *G* terms in Eq 25 in the present formulation differs from the covariance measurement update in a traditional extended Kalman filter formulation to account for the error-state mean reset (to zero) after each measurement update. The above process of prediction and measurement updates (when available) repeats each time step.

Next, we present four measurement models. The first two pertain to known kinematic states of the body segments (e.g., when the IMU is still) and the second two pertain to known kinematic constraints (e.g., constraints imposed by the two joints). Note that in the case of multiple measurements during a time step, a batch measurement update is used (i.e., all measurements are stacked and processed together).

Measurement model 1: ZUPT correction. We leverage the fact that a foot will be momentarily at rest sometime during the stance phase during gaits that do not induce significant slipping. Thus we employ, a zero-velocity update (ZUPT) correction for estimating foot trajectories which accurately describes gaits at normal walking speeds [13,16] through fast walking and running speeds [32]. Within our framework, the associated measurement equation becomes

$$h_{ZUPT}(x)=v_{IMU}$$ (28)

where *h*_*ZUPT*_*(x)* is the expected measurement for the ZUPT correction and *v*_*IMU*_ is the (3x1) velocity vector for a foot-mounted IMU. This expected foot velocity is compared to the (virtual) observed measurement of the foot velocity

$$z_{ZUPT}=\left[\begin{array}{c} 0\\ 0\\ 0 \end{array}\right]$$ (29)

when the IMU (foot) is (momentarily) still.

Measurement model 2: Gravitational tilt correction. We also leverage the fact that when an IMU is still, the accelerometer in the IMU measures only gravitational acceleration and therefore functions as an inclinometer, thus enabling a gravitational (tilt) correction for IMU orientation. This correction yields the measurement model

$$h_{tilt}(x)=R^{T}\left[\begin{array}{c} 0\\ 0\\ 1 \end{array}\right]$$ (30)

where *h*_*tilt*_*(x)* is the expected measurement for the gravitational correction and *R* is the rotation matrix for the still IMU. Note that in Eq 30, it is assumed that gravity acts opposite the third world-frame component (i.e., in the “*–z”* direction); however, this equation can be easily modified to accommodate other world-frame definitions. This expected measurement is compared to the observed measurement of tilt

$$z_{tilt}=\frac{a}{\left\|a\right\|}$$ (31)

with *a* being the IMU acceleration. Note that we compare the direction of unit vectors (*h*_*tilt*_ and *z*_*tilt*_) rather than the full acceleration vector to mitigate the effects of discrepancies in magnitude caused by the IMU not being exactly still or the effects of sensor noise and bias.

Measurement model 3: Joint center correction. Next, the joint center between two adjacent limbs must be approximately at the same position as deduced from the positions and orientations of those limbs [31]. For IMUs on adjacent limbs 1 and 2, the measurement equation becomes

$$h_{JC}(x)=p_{1}+R_{1}r_{1}-(p_{2}+R_{2}r_{2})$$ (32)

where *h*_*jc*_*(x)* is the expected measurement for the joint center correction, the subscript *i = 1*,*2* denotes *IMU*_*i*_, *r*_*i*_ denotes the known position of the joint center from *IMU*_*i*_ (and resolved in the IMU frame), and *R*_*i*_ denotes the rotation matrix for *IMU*_*i*_. The (virtual) observed measurement for the joint center correction is

$$z_{JC}=[\begin{array}{c} 0\\ 0\\ 0 \end{array}].$$ (33)

Measurement model 4: Joint axis correction. Similar to the joint center correction above, at times the joint axis must be the same as deduced from the orientations of the adjacent limbs (IMUs). An example of this correction arises when the knee is predominantly acting like a hinge [17,18] and the flexion/extension axes of the thigh and shank must be aligned in the world frame. This can be generalized for any pair of adjacent limbs 1 and 2 per

$$h_{JA}(x)=R_{1}e_{1}-R_{2}e_{2}$$ (34)

where *h*_*JA*_*(x)* is the expected measurement for the joint axis correction and *e*_*i*_ is the aligned joint axis (unit vector) deduced from *IMU*_*i*_ in the frame of *IMU*_*i*_. The (virtual) observed measurement for the joint axis correction is

$$z_{JA}=[\begin{array}{c} 0\\ 0\\ 0 \end{array}].$$ (35)

### Evaluation of ErKF method using two reference data sets

We evaluate the performance of the ErKF method using two sets of reference data, namely: 1) simulated IMU data for the simulated walker with associated simulated ground truth results and 2) experimental IMU data for the physical walker with associated MOCAP results. These two data sets allow us to evaluate the performance of the method with increasing levels of model complexity and uncertainty (e.g., knowledge of sensor noise characteristics, sensor-to-segment alignment) and without several confounding error sources from human subjects that affect both the ErKF method and reference MOCAP estimates including uncertainty in sensor-to-segment alignment parameters (e.g., relationships between sensor and anatomical coordinate systems, joint center locations), increased joint complexity, and soft tissue artefacts. To this end, we compare estimated and reference hip joint angles, stride length, and step width for the walker simulation and experiment (i.e., reference data sets 1 and 2 above). The reported hip joint angles mirror the International Society of Biomechanics convention for human subjects [33] with body axes defined such that the x-axis points anteriorly, the y-axis superiorly, and the z-axis to the right (aligned with hinge joint axis) when the model is in a neutral upright pose (as in Fig 1B). The definitions of stride length and step width parameters are consistent with [24] with the following minor modifications: 1) identified footfall instances are used in place of initial contact times, and 2) the IMU position is used in place of the heel position for the 3-body model. S1 Appendix details the methods for detecting footfalls and still periods. Further details for each reference data set are provided next.

Reference data set 1: ErKF method estimates for walker compared to simulation. We first evaluate the performance of the ErKF method via simulation because simulation enables assessment of the ErKF method independent of many confounding factors associated with experimental data. For example, in the simulation we specify the 1) sensor error (e.g., bias, noise) parameters, 2) sensor-to-segment alignment parameters, 3) measurement times (e.g. when a foot is stationary, when a joint acts as a hinge), and 4) ground truth data for comparison. We first compute generalized coordinate trajectories for a straight-line walk for the three body segments. The gait consists of 200 identical strides with a mean speed and stride length of 0.33 m/s and 0.73 m, respectively. Stance and swing angular trajectories were chosen to have a waveform similar to the simplest walking model [34]. The gait also contains (0.1 second) still periods following each ground contact, permitting clear identification of times of zero-velocity of the “feet”. The OpenSim model is then driven with these computed trajectories and the BodyKinematics analysis tool in OpenSim computes the virtual IMU poses with respect to a fixed lab-frame. Simulated IMU data (accelerations and angular rates) that are free of noise and bias are calculated by differentiating these poses at a sampling rate of 512 Hz (sampling rate of the IMUs used for *Reference Data Set 2*). Finally, real (i.e., noisy) IMU data is simulated by adding prescribed zero-mean Gaussian noise to this data. The accelerometer noise value is taken from the specification sheet for the commercial IMU (Opal, APDM, ±16 g and ±200g accelerometers, ±2000 deg/s gyro) used in the experiments. The gyro noise is that from the same specification sheet plus additional noise (10 deg/hr drift) to account for both bias instability and angular random walk. The joint center measurement noise comes from [23]. All noise values are summarized in Table 1.

**Table 1:** IMU and measurement noise values used for simulating data and for ErKF method.

Noise Parameter	σ_a_ (m/s^2^)	σ_*ω*_ (deg/s)	σ_ZV_ (m/s)	σ_tilt_ (deg)	σ_JC_ (m)	σ_JA_ (rad)
Value	0.027	5.66	0.01	5.73	0.01	1.15

Noise values for the process model and the simulated IMU data are for the accelerometer (σ_a_) and gyroscope (σ_*ω*_). Measurement noise values are for the zero-velocity (σ_ZV_), gravitational tilt (σ_tilt_), joint center (σ_JC_), and joint axis (σ_JA_) measurements.

Poses of each segment throughout the walk are estimated by employing the ErKF method with the simulated IMU data. Because the joints are constrained to be pure flexion/extension, the joint axis and joint center measurement corrections are applied at each time step with constant joint centers and joint axes. The segment poses follow directly from the IMU poses because the sensor-to-segment alignment is constant throughout the trial. Additionally, for this simulation, the sensor-to-segment alignment is known exactly.

Zero-velocity measurements are applied at identified footfalls while gravitational correction measurements are applied at each still period while using the measurement noise reported in Table 1. The accuracy of ErKF estimated hip joint angles, stride lengths, and step widths are compared to prescribed values from the original gait trajectories.

Reference data set 2: ErKF method estimates for walker compared to MOCAP. Next, we evaluate the performance of the method on the walker during overground walking gait. A marker-based motion-capture (MOCAP) system (Vicon, 18 Vero V2.2 cameras) tracks positions of reflective markers on the model at 100 Hz. Seven reflective markers are attached to each segment (four to define the primary axes and three additional markers, see Fig 1B). Positional estimates of the markers are filtered with a 4^**th**^ order low-pass Butterworth filter at 20 Hz. Additionally, the attached IMUs yield sampled acceleration and angular rate data at 512 Hz.

IMU poses on the segment (for sensor-to-segment alignment) are defined by three reflective markers attached to each IMU (Fig 1B). Body frame axes for the three segments are determined as described in *Reference data set 1*. Joint center locations are estimated at the center of the T-joints at the hips using the known dimensions of the model. A single still frame at the beginning of the trial is used to determine the positions of joint centers, IMUs, and reflective markers in the body-fixed frames and are assumed constant. MOCAP estimates of segment orientations are determined as follows using a published optimization method [35]. For each segment and time step, we record all pairwise positions between the markers on the segment and compare them to the same from the still frame. A MATLAB implementation of the aforementioned optimization method [36] is used to estimate segment orientation. Segment orientation estimates that yield (mean residual) marker positional errors exceeding 0.01 meters are eliminated as they indicate misidentified markers or significant marker positional error. Short time gaps (<0.05 seconds) in the orientation estimates are filled using linear interpolation for the resulting Euler angles (“unwrapped” to account for discontinuities) following which a 4^th^ order low-pass Butterworth filter (20 Hz cut-off) is applied for data smoothing. Rotational alignment between segment axes and their associated IMU sense axes is computed using a singular value decomposition procedure [37] comparing body-fixed IMU (from gyro measurement) to segment (from MOCAP segment orientation estimate) angular velocity vectors.

During the trial, a researcher manually operates (walks) the model back and forth through the 4.5-meter capture volume for ten minutes. The average sacrum velocity during straight walking is 0.44 m/s. Because the model is restricted to pure flexion/extension of the hip, joint center measurements are applied at every time step. Additionally, joint axis measurements are applied at every time step recognizing that the body-fixed z-axes (flexion/extension axes) of the pelvis and legs are aligned. As with the simulation, zero-velocity measurements are applied at all footfall instances and gravitational tilt measurements applied at all still periods. Measurement noise parameters used in the ErKF for data set 2 are the same as for data set 1 (Table 1).

## Results

### Reference data set 1: ErKF method estimates for walker compared to simulation

We open by comparing estimated outputs from the walker model (using simulated IMU data) to ground truth from the OpenSim simulation. Comparisons are made for all full strides excluding the first (transition) stride for each leg (198 strides for the right leg, 199 strides for the left leg). Joint angle estimates from the model are compared to ground truth values at each sample throughout the trial. Mean, standard deviation (SD), and root-mean-square (RMS) differences for the three joint angles are summarized in Table 2. RMS differences are specifically included for comparisons to results from [23,24]. Additionally, range of motion (ROM) is estimated and compared for each stride. Summary statistics for ROM differences are also reported in Table 2 for flexion/extension but not for internal/external rotation or abduction/adduction as their true values are constantly zero for this model.

**Table 2:** Comparisons of joint angle and range of motion estimates for simulation.

	Mean Diff. ±SD (deg)	RMS Diff. (deg)	Mean ROM Diff. ±SD (deg)	ROM RMS Diff. (deg)
Flexion/Extension	-0.01 ± 0.17	0.17	0.18 ± 0.23	0.29
Internal/External	0.00 ± 0.09	0.09	NR	NR
Abduction/Adduction	0.00 ± 0.08	0.08	NR	NR

Mean ± one standard deviation (SD) and root-mean-square (RMS) differences (IMU-true) in estimated hip joint angles and ranges of motion (ROM). Values reported across both hips (NR denotes not reported).

**Fig 2:**
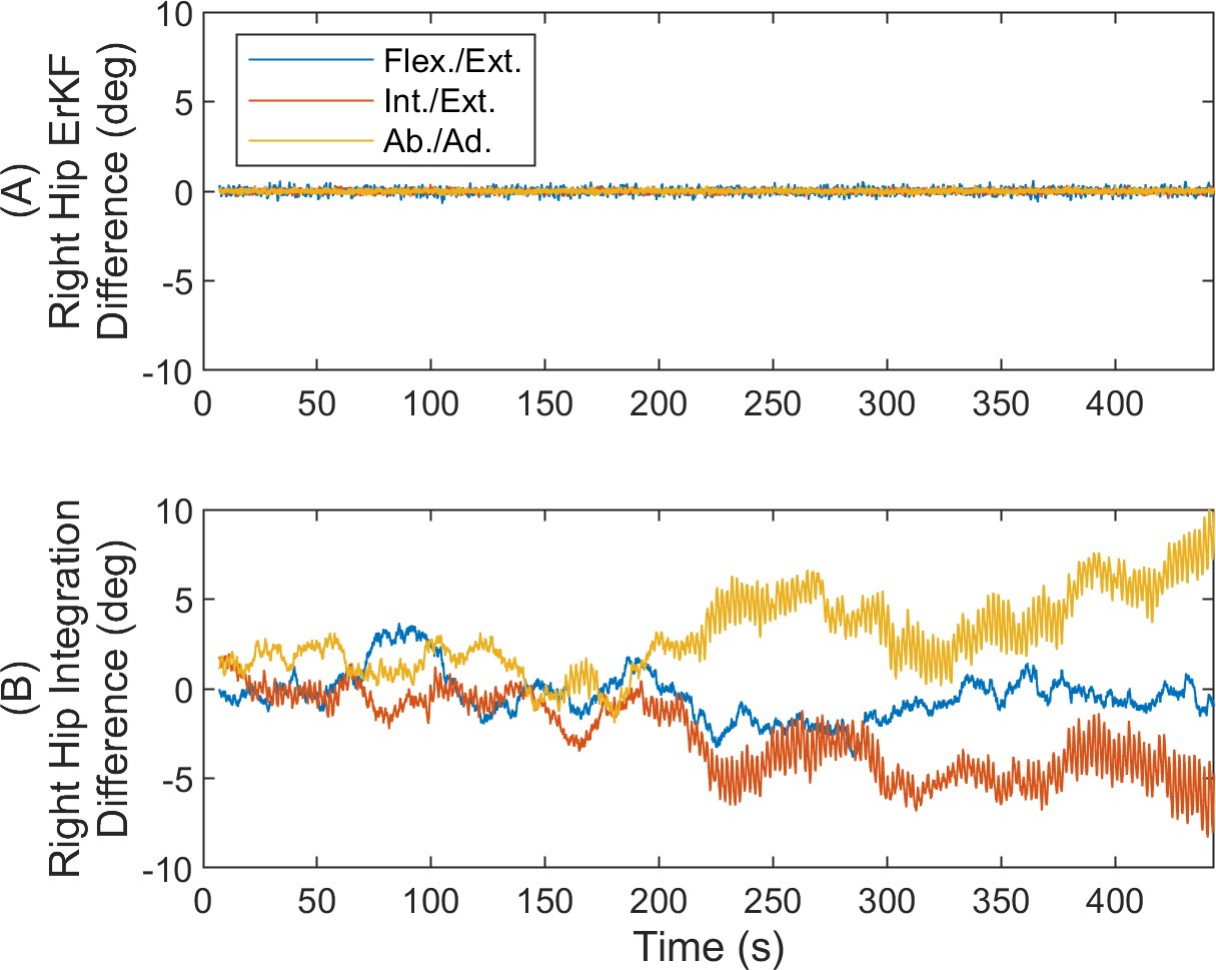
Joint angle differences with and without filtering corrections for simulation. Right hip joint angle differences versus time (A) with the ErKF corrections and (B) without any filtering corrections (raw integration). Hip angles are for flexion/extension (Flex./Ext.), internal/external rotation (Int./Ext.), and abduction/adduction (Ab./Ad.). Results reveal no observable drift error despite the long trial with ErKF method.

Fig 2 shows the differences in the three joint angles as functions of time for the right hip joint over this exemplary long (7 minute) trial (results similar for left hip). Importantly, the results reveal no observable drift in the joint angle differences with time (slopes of linear fits of the joint angle differences versus time remain below 0.1 deg/hr across all joint angles). By contrast, without any filter corrections, the differences can grow to up to 10 degrees due to drift over this same time interval.

**Fig 3:**
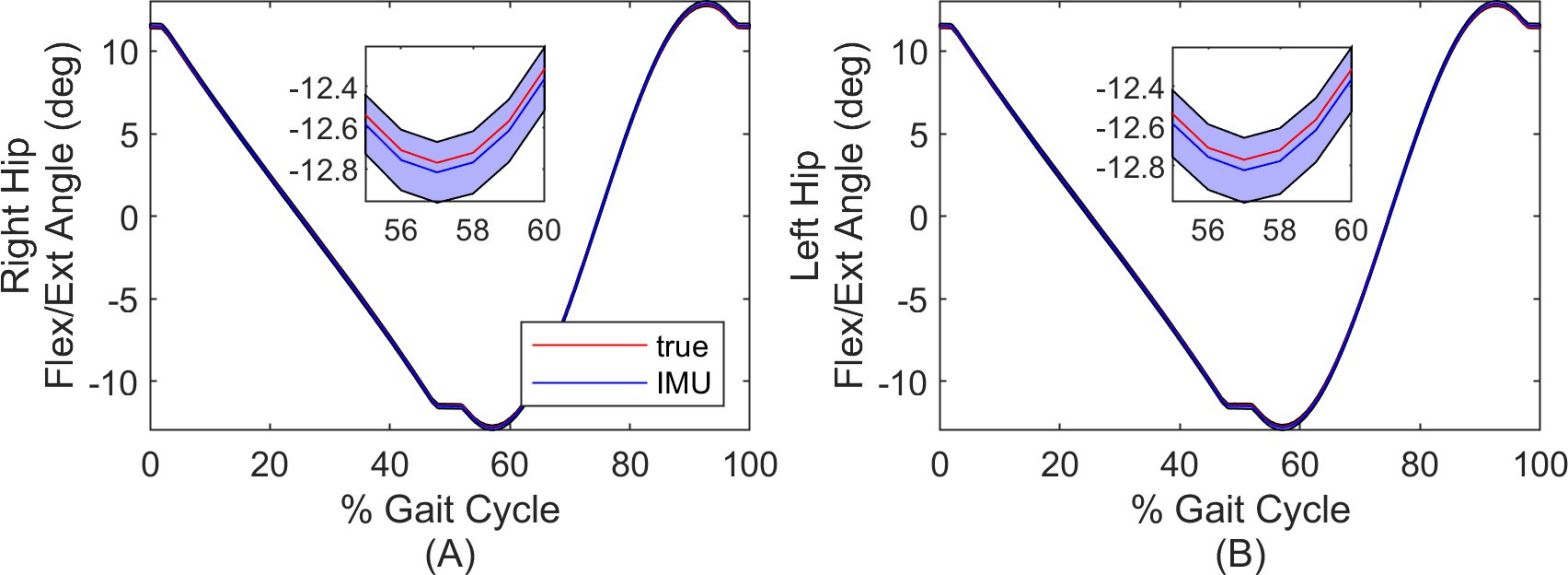
Comparisons of hip flexion/extension estimates for simulation. Mean and standard deviation of the estimated hip flexion/extension angle for the right (A) and left (B) hip. Solid lines denote mean and shaded regions denote ± one standard deviation. Time is normalized by gait cycle time. Insets provide zoomed images where small differences are apparent.

Fig 3 illustrates the estimated flexion/extension angle compared to ground truth during the gait cycle where time is normalized by gait cycle time (which begins and ends with the instances of identified footfalls). Shown are the average (solid line) and one standard deviation (shaded region) across all strides.

We also report the accuracy of the estimated stride length and step width as summarized in Table 3. The mean differences are less than 1% of the average values for both stride length (0.73 m) and step width (0.39 m), while the RMS differences remain within 1% and 4% respectively.

**Table 3:** Comparisons of stride length and step width estimates for simulation.

	Mean Diff. ±SD (m)	RMS Diff. (m)
Stride Length	0.01 ± 0.01	0.01
Step width	0.00 ± 0.01	0.01

Mean ± one standard deviation (SD) and root-mean-square (RMS) differences (IMU-true) in estimated stride length and step width for simulation.

**Fig 4:**
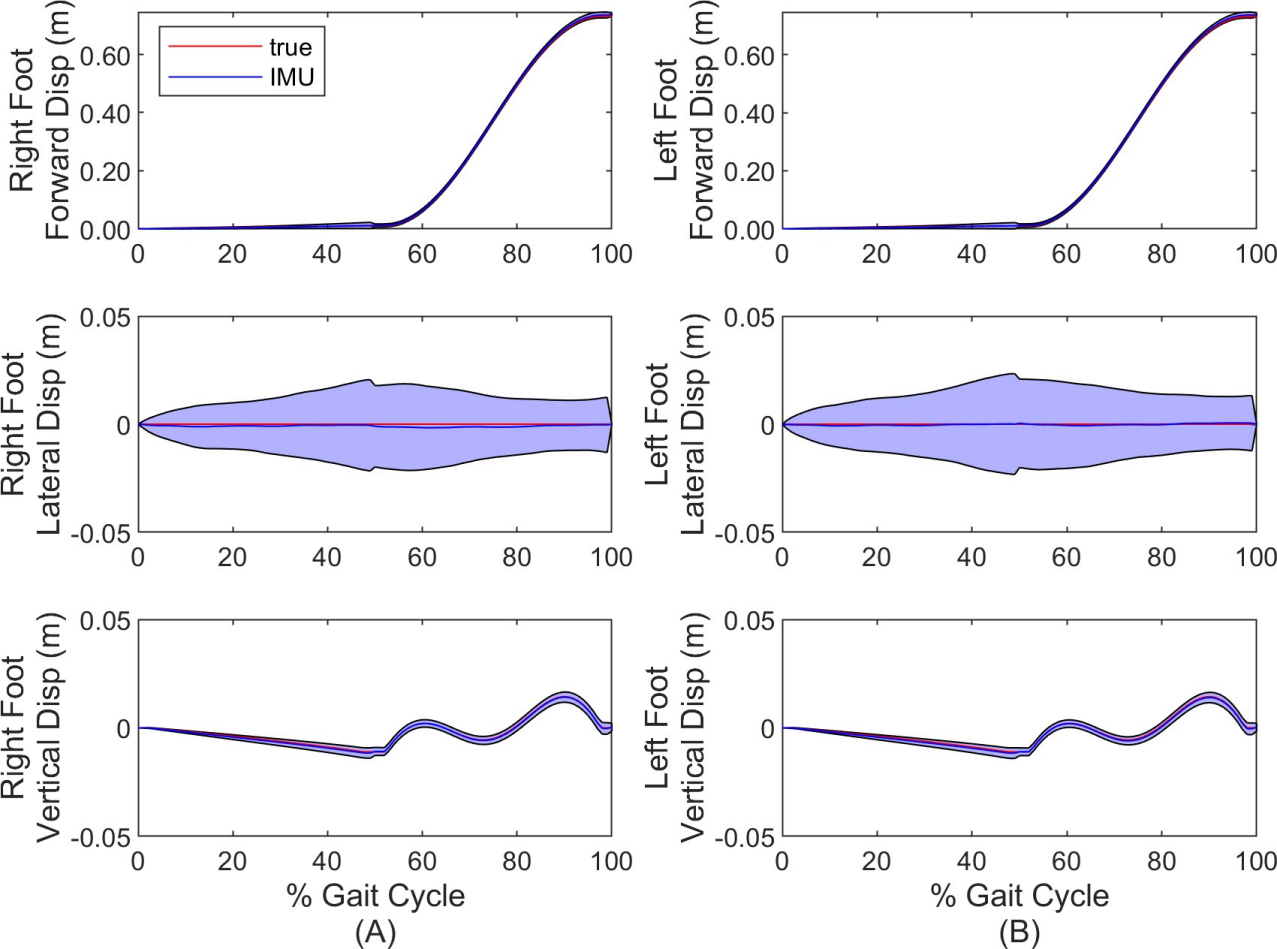
Comparisons of foot displacement estimates for simulation. Forward, lateral and vertical coordinates of right (A) foot and left (B) foot compared to ground truth. Solid lines denote the mean and shaded regions denote ± one standard deviation. Note significantly enlarged scales of lateral and vertical displacements.

Finally, we report the accuracy of the estimated foot IMU trajectories for each leg for the duration of the stride cycle. Fig 4 compares the forward, lateral and vertical coordinates of the right (Fig 4A) and left (Fig 4B) foot IMU to ground truth. Both mean (solid line) and one standard deviation (shaded region) are illustrated as functions of time (normalized by gait cycle). Note significantly enlarged scales for lateral and vertical displacements. Additionally, note that these trajectories show the relative displacement of the IMU center and not the position of the ground contact point itself (refer to Fig 1A); thus, negative vertical displacement (Fig 4) does not necessarily represent penetrations of the ground.

### Reference data set 2: ErKF method estimates for walker compared to MOCAP

Next, we compare estimated outputs from the model using measured IMU data to those measured by MOCAP. The results below report the differences in estimated kinematical quantities obtained by the two measurement modalities. Recall that the experimental procedure requires repeated walking through the MOCAP capture volume and thus consists of straight walks through the capture volume separated by sharp turns. Since the sharp turns do not represent human-like gait, we focus our evaluation only on the “straight walking strides”. A straight walking stride is defined as one where the total displacement of the foot during the stride is no more than fifteen degrees from the primary direction of travel and the stride length is greater than 0.2 meters. We also exclude from analysis the transition stride following a turn for each leg.

**Fig 5:**
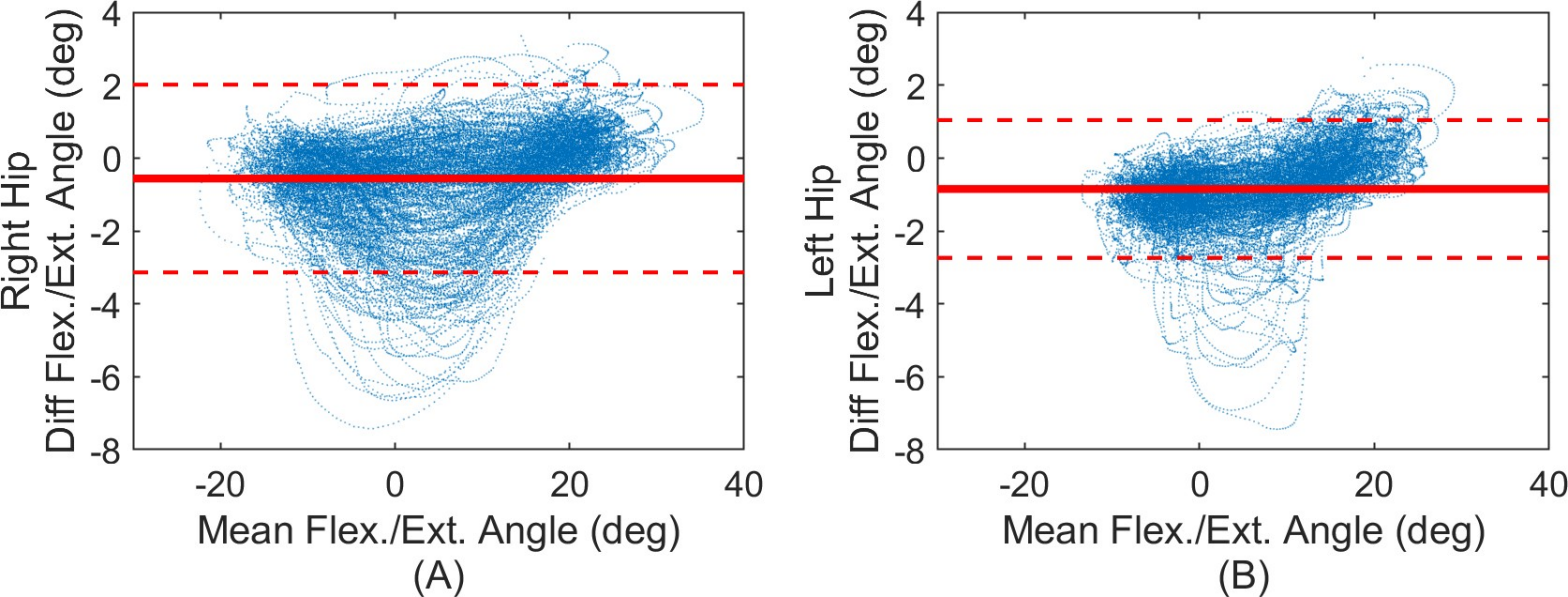
Bland-Altman plots of hip flexion/extension angle between IMU and MOCAP methods. Estimates for right (A) and left (B) hips. Blue points denote all samples, solid red line denotes the mean difference (IMU-MOCAP), and the red dashed lines denote 95% limits of agreement (LoA).

Consider first the differences in joint angle estimates from the IMU-based and MOCAP-based methods using data from the straight walking strides (239 strides for the right leg, 215 strides for the left leg). Bland-Altman plots of Fig 5 [38] illustrate the 95% limits of agreement (mean difference ± 1.96 times the standard deviation) between the two measurement modalities for the hip flexion/extension angle. Since the motion induces pure flexion/extension, we do not report Bland-Altman plots for internal/external rotation and abduction/adduction angles as the reference values are expected to be nominally zero. The mean differences for flexion/extension remain less than 1 degree for both hips with limits of agreement less than 3.2 degrees across both hips.

The mean, SD, and RMS differences for all three joint angles and the ROM difference for flexion/extension are reported in Table 4. As with the Bland-Altman plots, we do not report range of motion results for internal/external rotation and abduction/adduction angles as the reference values are expected to be nominally zero.

**Table 4:** Comparisons of joint angle and range of motion estimates for experiment.

	Mean Diff. ±SD (deg)	RMS Diff. (deg)	Mean ROM Diff. ±SD (deg)	ROM RMS Diff. (deg)
Flexion/Extension	-0.70 ± 1.17	1.36	0.85 ± 1.06	1.36
Internal/External	-0.39 ± 0.29	0.48	NR	NR
Abduction/Adduction	0.14 ± 0.56	0.58	NR	NR

Mean ± one standard deviation (SD) and root-mean-square (RMS) differences (IMU-MOCAP) in estimated hip joint angles and ranges of motion (ROM). Values reported across both hips (NR denotes not reported).

**Fig 6:**
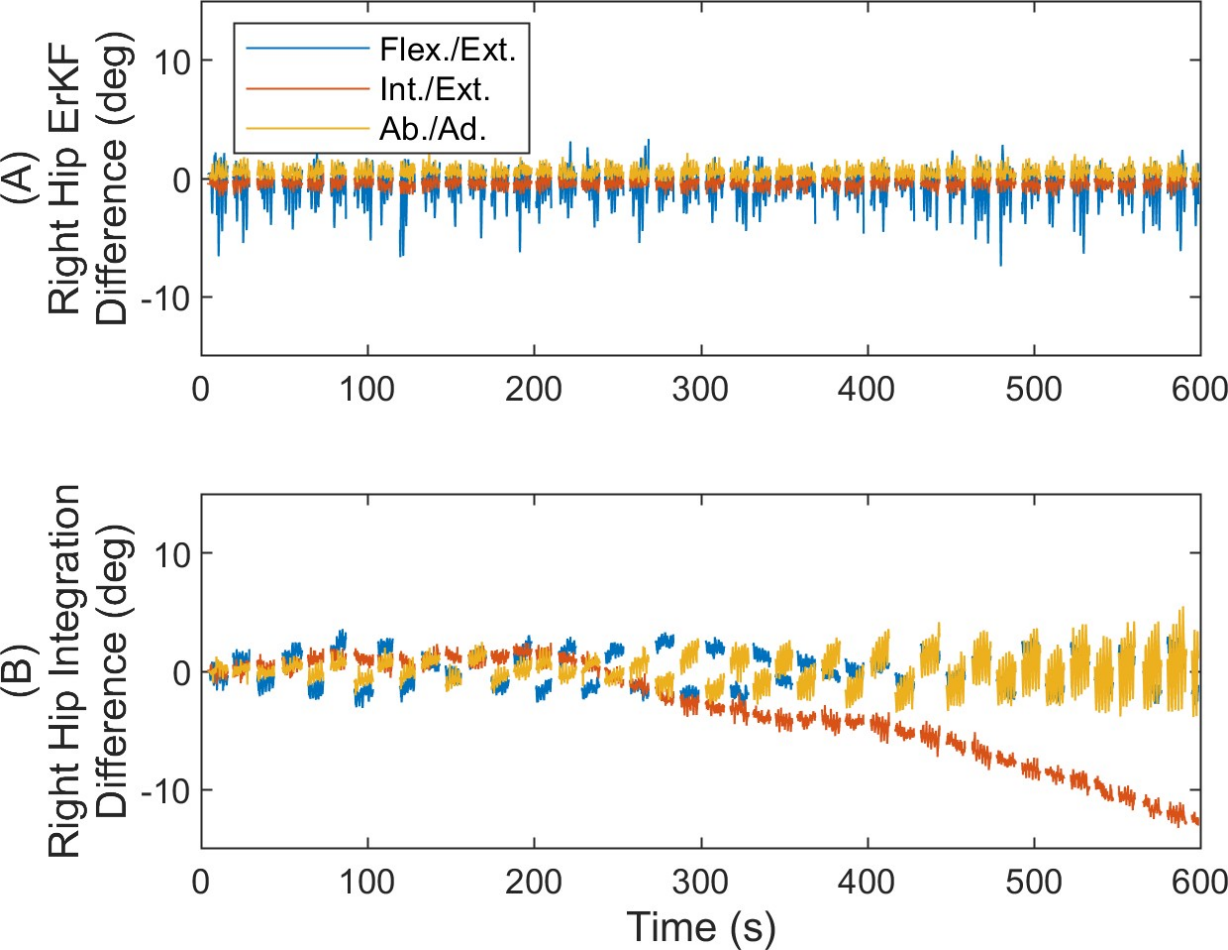
Joint angle differences with and without filtering corrections for experiment. Right hip joint angle differences versus time for all straight walking strides (A) with the ErKF corrections and (B) without any filtering corrections (raw integration). Hip angles are for flexion/extension (Flex./Ext.), internal/external rotation (Int./Ext.), and abduction/adduction (Ab./Ad.). Results reveal no observable drift error despite the long trial with ErKF method.

Next, we evaluate how the differences in estimated joint angles vary with time over the entire ten-minute trial. Fig 6 illustrates the right hip joint angle differences versus time for all straight walking strides (similar results for left hip). While very small biases between the two joint angle estimates exist, the results reveal no observable drift in the differences over the ten-minute trial (slopes of linear fits of the joint angle errors versus time remain below 1.8 deg/hr across all joint angles). By contrast, without any filter corrections, the differences can grow to up to 13 degrees due to drift over the 10-minute trial.

**Fig 7:**
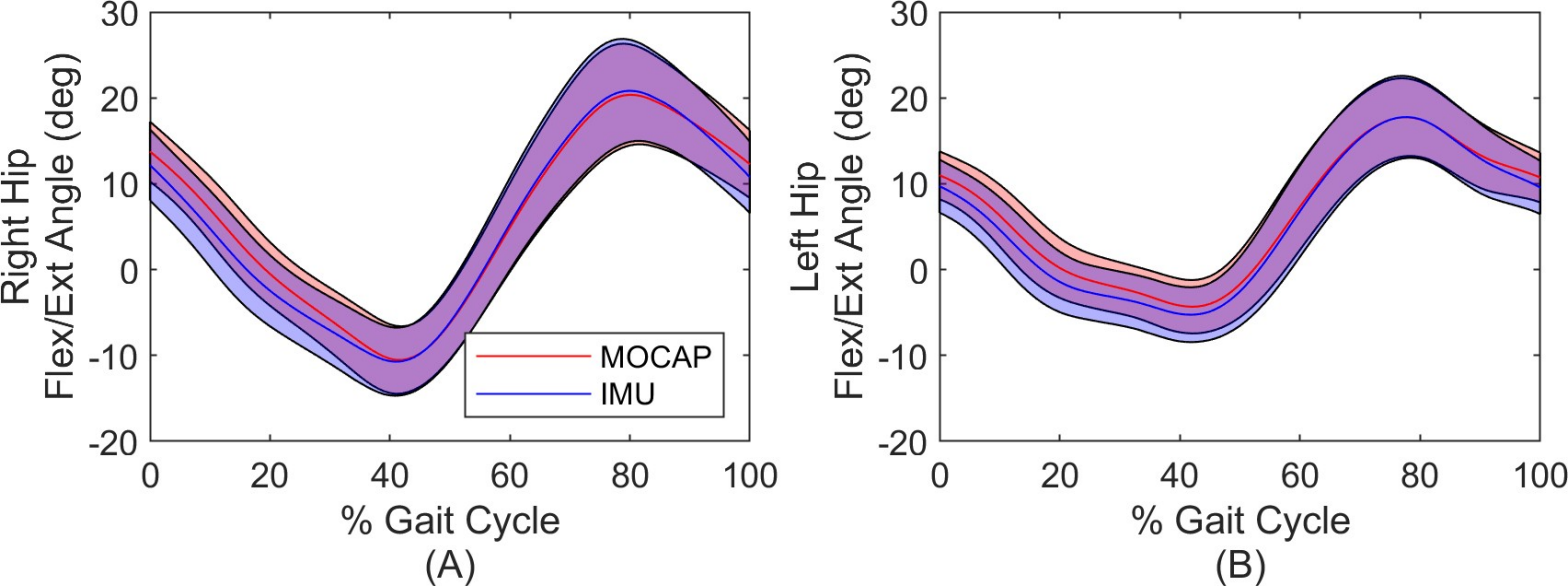
Comparisons of hip flexion/extension estimates for experiment. Mean and standard deviation of the estimated hip flexion/extension angle for the right (A) and left (B) hip. Solid lines denote mean and shaded regions denote ± one standard deviation. Time is normalized by gait cycle time.

Consider next a comparison of the flexion/extension angle estimates through the gait cycle as reported in Fig 7, following the same procedure described above in the context of Fig 3. Illustrated are the mean (solid curves) and one standard deviation from the mean (shaded regions) for both measurement modalities. The largest differences in the means arise during the stance phase. However, the measured stride-to-stride variability in flexion/extension angle (the average width of the shaded regions) is only 0.2 degrees larger for the IMU-based estimates versus MOCAP estimates.

Finally, we assess the differences in estimated stride length, step width and the trajectories of both feet for all straight walking strides. The differences in estimated stride length and step width over all strides are reported in Table 5. The mean differences remain below 2% of the average value for both stride length (0.77 m) and step width (0.38 m), while the RMS differences remain below 7% and 5%, respectively.

**Table 5:** Comparisons of stride length and step width estimates for experiment.

	Mean Diff. ±SD (m)	RMS Diff. (m)
Stride Length	0.01 ± 0.05	0.05
Step width	0.01 ± 0.02	0.02

Mean ± one standard deviation (SD) and root-mean-square (RMS) differences (IMU-MOCAP) in estimated stride length and step width for experimental model comparison.

**Fig 8:**
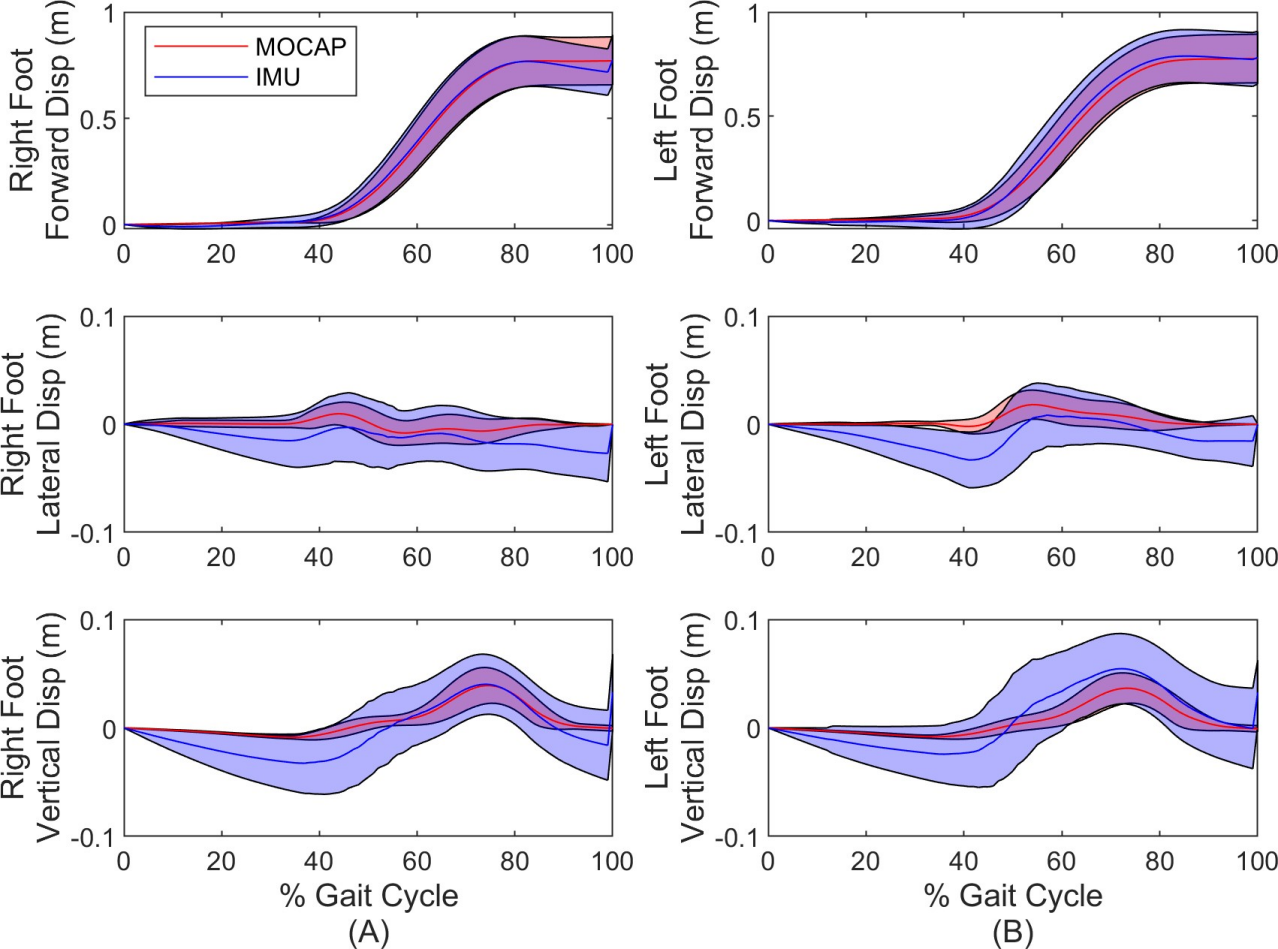
Comparisons of foot displacement estimates for experiment. Forward, lateral and vertical coordinates of right (A) foot and left (B) foot compared to MOCAP. Solid lines denote the mean and shaded regions denote ± one standard deviation. Note significantly enlarged scales of lateral and vertical displacements.

Fig 8 compares the forward, lateral and vertical coordinates of the right (Fig 8A) and left (Fig 8B) foot IMU to those measured by MOCAP. Both mean (solid line) and one standard deviation (shaded region) are illustrated as functions of time (normalized by gait cycle). Note significantly enlarged scales for lateral and vertical displacements. Additionally, note that these trajectories show the relative displacement of the IMU center and not the position of the ground contact point itself (refer to Fig 1B); thus, negative vertical displacement (Fig 8) does not necessarily represent penetrations of the ground.

## Discussion

This paper presents an IMU-based method that accurately estimates the kinematics of a simplified 3-body model of the human lower limbs for overground walking. The estimation method, developed using an error-state Kalman filter, fuses acceleration and angular rate data from three independent IMUs (one per rigid body) using four kinematic constraints. The kinematic constraints capture 1) foot zero-velocity updates, 2) gravitational tilt corrections, 3) joint center corrections and 4) joint axis corrections. The model is tested using two sets of comparison data, namely: 1) simulated IMU data from a simulated walker that yields ground truth results, and 2) experimental IMU data from a physical walker with associated MOCAP results.

Results using simulated IMU comparison data demonstrate the success of the underlying IMU-based estimation model when all required inputs (e.g., noise parameters, sensor-to-segment alignments) are known and when critical measurement times (e.g., zero-velocity times) are also known. Under these conditions, the hip angle estimates remain highly accurate (RMS difference below 0.2 degrees) and without detectable drift error despite long-duration trials (~7 min). Additionally, stride length and step width estimates exhibit very small differences (RMS less than 1% and 4% respectively) when compared to ground truth values.

The experimental results for the second comparison data set with an experimental walker demonstrate excellent agreement between the IMU and MOCAP-based estimates for the hip joint angles, stride length, step width, and foot trajectories. In particular, the IMU and MOCAP-based estimates of the hip joint angles exhibit limits of agreement less than 3.2 degrees for the flexion/extension angle with RMS differences less than 1.4 degrees across all three hip angle axes. Additionally, RMS differences for stride length and step width remain below 7% and 5% respectively, compared to nominal values. Importantly, the differences in all kinematic variables did not appear to drift despite the longer duration trial (~10 minutes). Because the differences neither drift nor increase with time, there is every reason to anticipate similarly tight error bounds over even longer time periods. Moreover, the IMU-based estimates exhibit similar variation in the flexion/extension angle compared to MOCAP estimates; refer to the similar spread of the shaded regions in Fig 7. In the absence of a prior study like ours on a mechanical walker, we instead compare this study to prior work evaluating IMU-based methods on humans, while also recognizing the limitations of such comparisons. Recall that Teufl et al. [23] estimate the joint angles of the human lower limbs from IMU data (via an iterated extended Kalman filter) during a 6-minute walking test for comparison to MOCAP results [23]. Consequently, results from this study might be compared to those of [23] by focusing on the knee joint in [23] as it acts predominantly like a hinge during walking (as does the hip joint in the 3-body model herein). Doing so reveals that the RMS differences (relative to MOCAP) reported in [23] (~1.5 degrees) is remarkably similar to that reported herein (1.37 degrees), although also acknowledging that this comparison is limited due to obvious differences between the 3-body model for a walker and a 7-body model for a human. Importantly though, the model herein removes limitations in [23] including the assumption of level ground. Consequently, the method herein may hold great promise in extending to a 7-body model for a human and particularly for applications where the level ground assumption does not hold, including during activities of daily living and for outdoor sports and exercise. Accurate results for this second comparison data set may also be affected by uncertainties in model inputs such as sensor-to-segment alignment and footfall and still period detection. We mitigate these uncertainties by using MOCAP data to establish sensor-to-segment alignment and to manually correct misidentified footfalls. While this yields a method that is not truly “MOCAP-free”, these topics (sensor-to-segment alignment and still period detection from IMU data alone) are themselves active areas of research for human applications [39–41].

The success of the novel ErKF method on a simplified mechanical model of the human lower limbs demonstrated in this study motivate its extension to a full (7-body) model. Extension to the full model for the human lower limbs presents many additional challenges including those due to complex human (versus mechanical) joints, uncertainties in model inputs (e.g., sensor-to-segment alignment, footfall detection), and soft tissue artefacts. Thus, future work must ensure that extensions of this ErKF method maintain accurate kinematic estimates despite these additional error sources. Importantly, future extensions should be evaluated against many different types of human gait, including abnormal gaits (e.g., due to injury or disease), to demonstrate its utility in a variety of clinical and other biomechanical applications. We note that the ErKF method developed here yields great promise for accurately estimating human lower-limb kinematics in such applications because it relies only on kinematic constraints that are largely independent of movement type.

## Supporting information

S1 Appendix Identification of footfalls and still periods. (PDF)

S1 File Results data file. (XLSX)

## References

[1] Sutherland DH. The evolution of clinical gait analysis: Part II kinematics. Gait Posture. 2002;16: 159–179. 10.1016/s0966-6362(02)00004-8 12297257

[2] Cavanagh PR, Kram R. Stride length in distance running: Velocity, body dimensions, and added mass effects. Med Sci Sports Exerc. 1989;21: 467–79. 10.1249/00005768-198908000-00020 2674599

[3] Ford KR, Myer GD, Toms HE, Hewett TE. Gender differences in the kinematics of unanticipated cutting in young athletes. Med Sci Sports Exerc. 2005;37: 124–129. 10.1249/01.MSS.0000150087.95953.C3 15632678

[4] Vitali R V., Cain SM, Ojeda L V., Potter M V., Zaferiou AM, Davidson SP, et al. Body-worn IMU array reveals effects of load on performance in an outdoor obstacle course. PLoS One. 2019;14: e0214008. 10.1371/journal.pone.0214008 30897123 PMC6428270

[5] Hamacher D, Singh NB, Van Dieën JH, Heller MO, Taylor WR. Kinematic measures for assessing gait stability in elderly individuals: A systematic review. J R Soc Interface. 2011;8: 1682–1698. 10.1098/rsif.2011.0416 21880615 PMC3203491

[6] Ojeda L V, Rebula JR, Kuo AD, Adamczyk PG. Influence of contextual task constraints on preferred stride parameters and their variabilities during human walking. Med Eng Phys. 2015;37: 929–936. 10.1016/j.medengphy.2015.06.010 26250066 PMC4604025

[7] Wang W, Adamczyk PG. Analyzing gait in the real world using wearable movement sensors and frequently repeated movement paths. Sensors (Switzerland). 2019;19: 1925. 10.3390/s19081925 31022889 PMC6515355

[8] Ranavolo A, Draicchio F, Varrecchia T, Silvetti A, Iavicoli S. Wearable monitoring devices for biomechanical risk assessment at work: Current status and future challenges—A systematic review. Int J Environ Res Public Health. 2018;15: 1–26. 10.3390/ijerph15092001 30217079 PMC6163390

[9] Pannurat N, Thiemjarus S, Nantajeewarawat E. Automatic fall monitoring: A review. Sensors (Switzerland). 2014;14: 12900–12936. 10.3390/s140712900 25046016 PMC4166886

[10] Wong C, Zhang Z-Q, Lo B, Yang G-Z. Wearable sensing for solid biomechanics: A Review. IEEE Sens J. 2015;15: 2747–2760. 10.1109/JSEN.2015.2393883

[11] Titterton DH, Weston JL. Strapdown Inertial Navigation Technology. IET Radar, Sonar, Navigation and Avionics Series. 2004. 10.1049/PBRA017E

[12] Sola J. Quaternion Kinematics for the Error-State KF. arXiv:1711.02508 [Preprint]. 2017. [cited 2019 Jan 3]. Available from: https://arxiv.org/abs/1711.02508.

[13] Ojeda L V, Borenstein J. Non-GPS navigation for security personnel and first responders. J Navig. 2007;60: 391–407. 10.1017/S0373463307004286

[14] Dissanayake G, Sukkarieh S, Nebot E, Durrant-Whyte H. The aiding of a low-cost strapdown inertial measurement unit using vehicle model constraints for land vehicle applications. IEEE Trans Robot Autom. 2001;17: 731–747. 10.1109/70.964672

[15] Weygers I, Kok M, Konings M, Hallez H, De Vroey H, Claeys K. Inertial sensor-based lower limb joint kinematics: A methodological systematic review. Sensors (Switzerland). 2020;20: 1–23. 10.3390/s20030673 31991862 PMC7038336

[16] Foxlin E. Pedestrian tracking with shoe-mounted inertial sensors. IEEE Comput Graph Appl. 2005;25: 38–46. 10.1109/mcg.2005.140 16315476

[17] Seel T, Raisch J, Schauer T. IMU-based joint angle measurement for gait analysis. Sensors (Switzerland). 2014;14: 6891–909. 10.3390/s140406891 24743160 PMC4029684

[18] Vitali R V, Cain SM, McGinnis RS, Zaferiou AM, Ojeda L V, Davidson SP, et al. Method for estimating three-dimensional knee rotations using two inertial measurement units: Validation with a coordinate measurement machine. Sensors. 2017;17. 10.3390/s17091970 28846613 PMC5620966

[19] Robert-Lachaine X, Mecheri H, Larue C, Plamondon A. Validation of inertial measurement units with an optoelectronic system for whole-body motion analysis. Med Biol Eng Comput. 2017;55: 609–619. 10.1007/s11517-016-1537-2 27379397

[20] Blair S, Duthie G, Robertson S, Hopkins W, Ball K. Concurrent validation of an inertial measurement system to quantify kicking biomechanics in four football codes. J Biomech. 2018;73: 24–32. 10.1016/j.jbiomech.2018.03.031 29602475

[21] Zhang JT, Novak AC, Brouwer B, Li Q. Concurrent validation of Xsens MVN measurement of lower limb joint angular kinematics. Physiol Meas. 2013;34. 10.1088/0967-3334/34/8/N63 23893094

[22] Ahmadi A, Destelle F, Unzueta L, Monaghan DS, Linaza MT, Moran K, et al. 3D human gait reconstruction and monitoring using body-worn inertial sensors and kinematic modeling. IEEE Sensors Journal. 2016. 10.1109/JSEN.2016.2593011

[23] Teufl W, Miezal M, Taetz B, Fröhlich M, Bleser G. Validity, test-retest reliability and long-term stability of magnetometer free inertial sensor based 3D joint kinematics. Sensors (Switzerland). 2018;18. 10.3390/s18071980 29933568 PMC6068643

[24] Teufl W, Lorenz M, Miezal M, Taetz B, Fröhlich M, Bleser G. Towards inertial sensor based mobile gait analysis: Event-detection and spatio-temporal parameters. Sensors (Switzerland). 2019;19: 38. 10.3390/s19010038 30583508 PMC6339047

[25] Delp SL, Anderson FC, Arnold AS, Loan P, Habib A, John CT, et al. OpenSim: Open-source software to create and analyze dynamic simulations of movement. IEEE Trans Biomed Eng. 2007;54: 1940–1950. 10.1109/TBME.2007.901024 18018689

[26] Jacobs DA, Seth A. Dynamic walking challenge: Go the distance! OpenSim Documentation. [cited 10 Mar 2021]. Available from: https://simtk-confluence.stanford.edu:8443/pages/viewpage.action?pageId=28777060.

[27] Trawny N, Roumeliotis SI. Indirect Kalman filter for 3D attitude estimation. Department of Computer Science and Engineering, University of Minnesota, Minneapolis, MN; 2005. Available from: http://mars.cs.umn.edu/tr/reports/Trawny05b.pdf.

[28] Madyastha VK, Ravindray VC, Mallikarjunan S, Goyal A. Extended Kalman filter vs. error state Kalman filter for aircraft attitude estimation. AIAA Guid Navig Control Conf 2011. 2011. 10.2514/6.2011–6615

[29] Hartley R, Ghaffari M, Eustice RM, Grizzle JW. Contact-aided invariant extended Kalman filtering for robot state estimation. Int J Rob Res. 2020;39: 402–430. 10.1177/0278364919894385

[30] Vitali R V., McGinnis RS, Perkins NC. Robust error-state Kalman filter for estimating IMU orientation. IEEE Sens J. 2020. 10.1109/jsen.2020.3026895

[31] Miezal M, Taetz B, Bleser G. On inertial body tracking in the presence of model calibration errors. Sensors (Switzerland). 2016;16: 1132. 10.3390/s16071132 27455266 PMC4969842

[32] Potter M V, Ojeda L V, Perkins NC, Cain SM. Effect of IMU design on IMU-derived stride metrics for running. Sensors (Switzerland). 2019;19: 2601. 10.3390/s19112601 31181688 PMC6603669

[33] Wu G, Siegler S, Allard P, Kirtley C, Leardini A, Rosenbaum D, et al. ISB recommendation on definitions of joint coordinate system of various joints for the reporting of human joint motion—Part I: Ankle, hip, and spine. Journal of Biomechanics. 2002. pp. 543–548. 10.1016/s0021-9290(01)00222-6 11934426

[34] Garcia M, Chatterjee A, Ruina A, Coleman M. The simplest walking model: Stability, complexity, and scaling. J Biomech Eng. 1998;120: 281–288. 10.1115/1.2798313 10412391

[35] Shuster MD, Oh SD. Three-axis attitude determination from vector observations. J Guid Control Dyn. 1981;4: 70–77. 10.2514/3.19717

[36] Lourakis M. Absolute orientation with the QUEST algorithm. MATLAB Central File Exchange. 2020 [cited 5 May 2020]. Available from: https://www.mathworks.com/matlabcentral/fileexchange/65173-absolute-orientation-with-the-quest-algorithm.

[37] Challis JH. A procedure for determining rigid body transformation parameters. J Biomech. 1995;28: 733–737. 10.1016/0021-9290(94)00116-l 7601872

[38] Bland JM, Altman DG. Measuring agreement in method comparison studies. Stat Methods Med Res. 1999;8: 135–160. 10.1177/096228029900800204 10501650

[39] Pacini Panebianco G, Bisi MC, Stagni R, Fantozzi S. Analysis of the performance of 17 algorithms from a systematic review: Influence of sensor position, analysed variable and computational approach in gait timing estimation from IMU measurements. Gait Posture. 2018;66: 76–82. 10.1016/j.gaitpost.2018.08.025 30170137

[40] Bouvier B, Duprey S, Claudon L, Dumas R, Savescu A. Upper limb kinematics using inertial and magnetic sensors: Comparison of sensor-to-segment calibrations. Sensors (Switzerland). 2015;15: 18813–18833. 10.3390/s150818813 26263993 PMC4570347

[41] Vitali R V., Perkins NC. Determining anatomical frames via inertial motion capture: A survey of methods. J Biomech. 2020;106: 109832. 10.1016/j.jbiomech.2020.109832 32517995

