# Supplementary material for: Error-state Kalman filter for lower-limb kinematic estimation: Evaluation on a 3-body model
Source: PLoS One. 2021 Apr 20;16(4):e0249577. doi: 10.1371/journal.pone.0249577 (PMC8057618; doi:10.1371/journal.pone.0249577)
Supplement: S1 Appendix — (PDF) [file pone.0249577.s001.pdf]

S1 Appendix: Identification of footfalls and still periods

Here, we detail how footfalls and still periods are determined for the study. Footfalls and still periods are times when the foot is assumed to be still, with only one footfall being identified per stride.

Using the methods outlined below, success in identifying a footfall compared to the reference measurement system (simulation or MOCAP) is >99% for the model. We manually correct missing and misidentified footfalls (using velocity estimates from the reference system) so that our results are not impacted by errors in footfall identification. We note that these specialized methods are specifically developed for the current application and may not translate well to human gait patterns.

Reference Data Set 1: Model Estimates for Walker Compared to Simulation

Footfalls are identified exactly in the middle of the prescribed 0.1 second still periods at the beginning of the stance phase. Still periods are identified as all instances where the entire model was still (0.1 seconds following each heel contact).

Reference Data Set 2: Model IMU Estimates for Walker Compared to MOCAP

Still periods and foot impacts are estimated as follows. For each foot IMU, angular velocity data is filtered using a 4th order low-pass Butterworth filter at 6 Hz. Angular acceleration is then calculated from the filtered angular velocity using finite differentiation. Still periods are determined when three criteria were simultaneously satisfied:

1) Angular acceleration magnitude < 115 deg/s^2
2) Angular velocity magnitude < 45 deg/s
3) |(Linear acceleration magnitude – 1g)| < 0.2 × g

where |x| denotes the absolute value of x and g denotes the gravitational acceleration. Foot impacts are identified as peaks in angular velocity magnitude that is high-pass filtered at 20Hz with a 4th order Butterworth filter.

Next, strides are segmented using the filtered angular velocity data. Specifically, peaks associated primarily with the internal rotation axis greater than 3 seconds apart identify when the subject is turning while peaks associated with the flexion axis greater than 1 second apart segment individual strides during walking. The footfall in each stride is then identified from still periods following the associated stride's foot impact. Specifically, the footfall is specified in the middle of the longest continuous series of still periods (excluding gaps 1 sample in length) that is within 0.3 seconds of a foot impact during that stride.
